# Artificial intelligence-based pathologic myopia identification system in the ophthalmology residency training program

**DOI:** 10.3389/fcell.2022.1053079

**Published:** 2022-11-03

**Authors:** Zhi Fang, Zhe Xu, Xiaoying He, Wei Han

**Affiliations:** ^1^ Department of Eye Center, The Second Affiliated Hospital, Zhejiang University School of Medicine, Hangzhou, Zhejiang, China; ^2^ Zhejiang Provincial Key Lab of Ophthalmology, Hangzhou, Zhejiang, China

**Keywords:** artificial intelligence, pathologic myopia, myopic maculopathy, “Plus” lesion, ophthalmology residency training

## Abstract

**Background:** Artificial intelligence (AI) has been successfully applied to the screening tasks of fundus diseases. However, few studies focused on the potential of AI to aid medical teaching in the residency training program. This study aimed to evaluate the effectiveness of the AI-based pathologic myopia (PM) identification system in the ophthalmology residency training program and assess the residents’ feedback on this system.

**Materials and Methods:** Ninety residents in the ophthalmology department at the Second Affiliated Hospital of Zhejiang University were randomly assigned to three groups. In group A, residents learned PM through an AI-based PM identification system. In group B and group C, residents learned PM through a traditional lecture given by two senior specialists independently. The improvement in resident performance was evaluated by comparing the pre-and post-lecture scores of a specifically designed test using a paired *t*-test. The difference among the three groups was evaluated by one-way ANOVA. Residents’ evaluations of the AI-based PM identification system were measured by a 17-item questionnaire.

**Results:** The post-lecture scores were significantly higher than the pre-lecture scores in group A (*p* < 0.0001). However, there was no difference between pre-and post-lecture scores in group B (*p* = 0.628) and group C (*p* = 0.158). Overall, all participants were satisfied and agreed that the AI-based PM identification system was effective and helpful to acquire PM identification, myopic maculopathy (MM) classification, and “Plus” lesion localization.

**Conclusion:** It is still difficult for ophthalmic residents to promptly grasp the knowledge of identification of PM through a single traditional lecture, while the AI-based PM identification system effectively improved residents’ performance in PM identification and received satisfactory feedback from residents. The application of the AI-based PM identification system showed advantages in promoting the efficiency of the ophthalmology residency training program.

## Introduction

Artificial intelligence (AI) models have shown equal or better performance in disease diagnosis and management based on the medical image, such as diabetic retinopathy (Ruamviboonsuk et al., 2022), glaucoma (Medeiros et al., 2021; Ibrahim et al., 2022), age-related macular degeneration (Yan et al., 2021; Potapenko et al., 2022), congenital cataract (Lin et al., 2019), central serous chorioretinopathy (Xu et al., 2021; Jin and Ye, 2022), and papilledema (Milea et al., 2020). AI-based teaching can improve students’ or junior residents’ performance and satisfaction during ophthalmology clerkship, especially showing an advantage in deepening understanding of signs and morphological features (Wu et al., 2020; Han et al., 2022). Previous surveys showed the majority of medical staff or ophthalmologists believed AI will improve the practice of ophthalmology and should be incorporated into medical school and residency curricula (Valikodath et al., 2021a; Valikodath et al., 2021b; Zheng et al., 2021).

With the rapid increase of myopia prevalence, the incidence in high myopia was significantly raised as well (Grzybowski et al., 2020). Myopic maculopathy (MM) is a group of severe sight-threatening complications among pathologic myopia (PM) patients, which usually need extensive examination and evaluation by retinal specialists. The meta-analysis of a pathologic myopia system (META-PM) defined PM and provided a photographic classification and grading system for MM (Ohno-Matsui et al., 2015). The morphological and functional characteristics in eyes with high myopia were positively correlated with the severity classified by METE-PM (Zhao et al., 2020; Li et al., 2021). However, it usually takes a long time to cultivate a qualified or experienced retinal specialist. Therefore, we are always facing a shortage of retinal specialists, especially in primary healthcare and community medical service institutes. It is also a heavy task to teach PM knowledge to relevant clinicians like residents of ophthalmology. The shortage of specialist manpower leads to the potential application of AI technology in clinical teaching and training tasks.

A series of deep learning systems were designed to detect PM and MM classification based on color fundus images with comparable performance to the general ophthalmologist and retinal specialist (Lu et al., 2021b). We further developed an AI-based system for automatic PM identification, MM classification, and “Plus” lesion detection based on retinal color fundus images (namely Ophthalmology-client), which achieved excellent accuracy (Lu et al., 2021a). AI-based models have been proven to be a potential resolution to aid diagnosis and classification based on fundus photography. In this study, we aim to evaluate the effectiveness of the AI-aided teaching model in a group of ophthalmology residents using our AI-based PM identification system. The performance of the AI system is also compared with that of the traditional lecture-based teaching model.

## Materials and methods

### Participant enrollment and assignment

Ninety residents participating in the ophthalmology residency training program at the Second Affiliated Hospital of Zhejiang University were enrolled in June 2022. The participants were randomly assigned into three groups (1:1:1 ratio) and parallelly finished the training on the same day. In group A, the residents were instructed to learn the PM knowledge through an AI-based PM identification system by exploring and operating an AI-aided diagnosis platform-Ophthalmology-client. In groups B and C, the residents learned PM fundus image features through the traditional lecture given by two senior specialists respectively. All the procedures in this study were arranged strictly with the approval of the ethics committee of the Second Affiliated Hospital, School of Medicine, Zhejiang University. Written informed consent was given by every participant.

**FIGURE 1:**
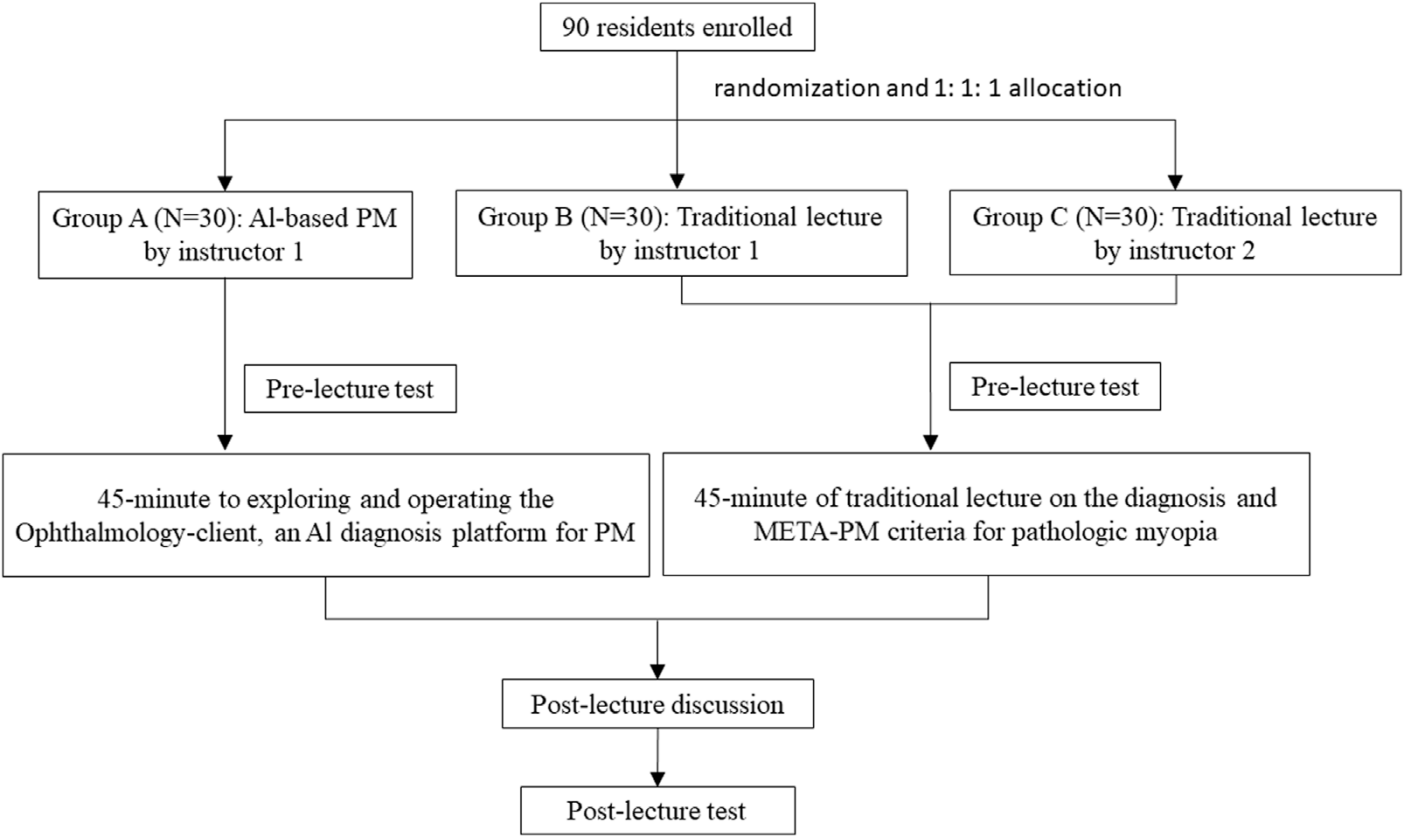
Flowchart of the study.

The flowchart of the study was shown in Figure 1. All three groups received a pre-lecture test, a 45-minute lecture, and a post-lecture test. The same specific designed test was used for pre-and post-lecture tests including three parts: part Ⅰ was the recognition of PM, part Ⅱ was the MM classification, and part Ⅲ was the “Plus” lesion detection.

Group A and group B were guided by an experienced instructor 1 (Dr. Zhi Fang). Group C was guided by another experienced instructor 2 (Dr. Zhe Xu) following the same working flow. Both instructors are physicians from the eye center of the Second Affiliated Hospital of Zhejiang University.

### Ophthalmology-client, the AI-aided diagnosis platform for PM

Ophthalmology-client is an AI–aided PM diagnosis and classification platform developed by our team. This AI platform can identify PM or non-PM, classify the MM, and detect “Plus” lesions based on retinal fundus images. The AI platform also can automatically localize the “Plus” lesions based on retinal fundus images with comparable performance to retinal experts after being trained with a large number of retinal fundus images.

### AI-based PM identification system

The residents in group A learned the contents including the introduction of Ophthalmology-client as well as the criteria for diagnosis of PM and the META-PM system 1 day before the class. Besides, the instructor encouraged them to discuss the key points of the retinal fundus image before the class. In the class, 10 min were given to allow the residents to finish the pre-lecture test. Then the residents had 45 min to explore and operate the AI-aided platform of Ophthalmology-client. The content of AI training regarding the PM topic included fundus images from a database on the website involving typical PM at different categories or non-PM and a report from the Ophthalmology-client. The contents of the output report included the category of PM according to the META-PM classification system if it is a PM, the location of the specific lesion with labels if there is a PM “Plus” lesion, and a brief introduction of PM. A 10-minute discussion regarding the key points and “Plus” lesions of PM fundus image was conducted. Finally, 10 min were given to finish the post-lecture test.

### Conduction of the traditional lecture

The residents in group B learned the knowledge of PM diagnosis and the META-PM system 1 day before class. The instructor encouraged them to discuss the key points of the retinal fundus image before the class. During the class, 10 min were given to residents to finish the pre-lecture test. Subsequently, the instructor gave a 45-minute traditional lecture on the topic of PM. The content of the traditional lecture regarding the PM topic included the brief introduction and typical fundus image of PM, the META-PM classification, and relevant typical fundus with the indication of chorioretinal atrophy, macular atrophy, and “Plus” lesion (lacquer cracks, choroidal neovascularization, or Fuchs spot). A 10-minute discussion regarding the key points and “Plus” lesions of PM fundus image was conducted. Finally, 10 min were given to finish the post-lecture test.

### Residents’ evaluations

The residents’ evaluations of the AI-based PM identification system were measured by a 17-item questionnaire, including 16 one-choice questions and one open-ended question (Table 1). The questionnaire in our study was designed based on previous studies of medical education, which rated on a 4-point scale ranging from “strongly agree” (with the highest score) to “strongly disagree” (with the lowest score) (Huang et al., 2016; Wu et al., 2020). The questionnaire was conducted in group A after class to collect and assess residents’ satisfaction with the AI-based PM identification system. Information of the questionnaire had several topics, including knowledge acquisition (2 items), motivational dimension (3 items), group cooperation (1 item), creative and critical thinking (1 item), instructor performance (3 items), organization (1 item), overall rating (2 items), recommendations (2 items).

**TABLE 1:** Seventeen-item questionnaire.

No.	Question
One-choice questions (A, strongly agree; B, agree; C, disagree; D, strongly disagree)	
1	AI-based PM identification system helped me to acquire a higher level of knowledge
2	AI-based PM identification system is more effective and motivate compared with traditional didactic lecture
3	AI-based PM identification system challenged me to do my best
4	AI-based PM identification system promoted the learning of essential concepts or skills
5	AI-based PM identification system promoted effective cooperative learning
6	AI-based PM identification system promoted increased reading of the textbook by the students
7	Overall, I am very satisfied with the AI-based PM identification system
8	AI-based PM identification system should be offered more frequently in the curriculum
9	I will recommend the AI-based PM identification system to other residents
10	This activity was preferable to the traditional lecture
11	AI-based PM identification system is easy to operate and well-designed
12	I study with colleagues frequently
13	The instructor highly facilitated the learning process of AI-based PM identification system
14	The instructor can well answer the residents’ questions
15	The instructor encouraged and provided opportunities for discussion
16	AI-based PM identification system is beneficial to help develop creative thinking and self-learning ability
17	Compared with the traditional teaching method, what do you think are the advantages and disadvantages of the AI-based PM identification system?

AI, artificial intelligence; PM, pathologic myopia.

### Data analysis

All data from the survey questionnaire were gathered by Questionnaire Star anonymously. We described categorical variables as frequencies and percentages. The effects of the AI-based platform on residents’ performance were measured by comparing pre-and post-lecture scores using a paired *t*-test. The difference among the three groups was evaluated by one-way ANOVA. Furthermore, an independent *t*-test was used to compare the improvement in performance between the three groups. A subgroup analysis was conducted between senior residents (3rd year) and junior residents (1st–2nd year) in both groups. It was considered statistically significant when *p* < 0.05. All data were analyzed by SPSS version 22.0 software (SPSS Inc., Chicago, IL, United States).

## Results

### Baseline characteristics

All participants finished the class and completed the pre-and post-lecture tests. There was no significant difference in the baseline characteristics between the three groups including gender, educational background, grade, and age (*p* = 0.610) (Table 2). The percentage of the male was 33.3%, 33.3%, and 30%; the percentage with a postgraduate degree was 66.7%, 63.3%, and 63.3%; the percentage of senior residents (3rd year) was 36.7%, 33.3%, and 30%; the age was 27.53 ± 3.48 years, 27.97 ± 3.06 years, and 27.10 ± 3.55 years respectively in groups A, B, and C.

**TABLE 2:** Baseline characteristics of participants.

Characteristics	Group A	Group B	Group C	*p* Value
Male (n, %)	10, 33.3%	10, 33.3%	9, 30%	
Postgraduate (n, %)	20, 66.7%	19, 63.3%	19, 63.3%	
3rd year resident (n, %)	11, 36.7%	10, 33.3%	9, 30%	
Age (years, mean ± SD)	27.53 ± 3.48	27.97 ± 3.06	27.10 ± 3.55	0.610

SD, standard deviation.

### Pre-lecture scores of residents’ performance in each group

The total and three parts of pre-lecture scores were similar between three groups with no significant difference (Table 3). Subgroup analysis showed there was no obvious difference in the total, part Ⅱ, and part Ⅲ of pre-lecture scores between the senior and junior residents. However, the pre-lecture scores of part Ⅰ were significantly higher among the senior residents’ than the junior residents (Table 4, *p* = 0.030).

**TABLE 3:** Pre-lecture scores of three groups.

	Group A (mean ± SD)	Group B (mean ± SD)	Group C (mean ± SD)	*p* Value
Part Ⅰ	15.43 ± 2.06	15.23 ± 1.92	14.93 ± 2.12	0.634
Part Ⅱ	7.67 ± 2.09	8.57 ± 2.31	8.33 ± 1.58	0.207
Part Ⅲ	12.20 ± 3.03	11.77 ± 2.77	11.70 ± 2.82	0.767
Total	35.30 ± 5.72	35.57 ± 5.22	34.97 ± 4.62	0.905

SD, standard deviation.

**TABLE 4:** Pre-lecture scores between junior and senior residents.

	Junior (1st–2nd year, mean ± SD)	Senior (3rd year, mean ± SD)	*p* Value
Part Ⅰ	14.83 ± 1.96	15.80 ± 1.95	0.030^*^
Part Ⅱ	7.95 ± 1.99	8.63 ± 2.08	0.133
Part Ⅲ	11.70 ± 2.84	11.93 ± 2.57	0.705
Total	34.48 ± 5.04	36.37 ± 5.01	0.097

*p < 0.05. SD, standard deviation.

### Improvement of residents’ performance in each group

One-way ANOVA detected significant difference in the total (*p* < 0.0001), part Ⅰ (*p* = 0.024), part Ⅱ (*p* < 0.0001), and part Ⅲ (*p* < 0.0001) of post-lecture scores among three groups. Further *t*-test found the total, part Ⅱ, and part Ⅲ of post-lecture scores were significantly higher than the pre-lecture scores in group A (Table 5, *p* < 0.01). However, we found no improvement of part Ⅰ in group A (Table 5, *p* = 0.199). In group B, the improvement was not obvious in total (*p* = 0.302), part Ⅰ (*p* = 0.087), and part Ⅱ (*p* = 0.504) (Table 5). In group C, the improvement was also not significant in total (*p* = 0.158), part Ⅰ (*p* = 0.808), and part Ⅱ (*p* = 0.594) (Table 5). However, significant improvement of part Ⅲ about the “Plus” lesion detection was observed in both group B and group C (Table 5, *p* < 0.05). The total, part Ⅰ, part Ⅱ, and part Ⅲ of post-lecture scores in group A were significantly higher than those in group B (Table 5, *p* < 0.01). However, there was no significant difference of post-lecture scores between group B and group C (Table 5, *p* = 0.489 for total scores, *p* = 0.340 for part Ⅰ scores, *p* = 0.375 for part Ⅱ scores, *p* = 0.137 for part Ⅲ scores), which indicated a similar effect of training by different instructors.

**TABLE 5:** Improvement of residents’ performance of three groups.

		Part Ⅰ	Part Ⅱ	Part Ⅲ	Total
Group A (mean ± SD)	Pre-lecture	15.43 ± 2.06	7.67 ± 2.09	12.20 ± 3.03	35.30 ± 5.72
	Post-lecture	16.00 ± 1.91^##^	14.27 ± 3.54^**##^	15.47 ± 1.80^**##^	45.73 ± 5.00^**##^
Group B (mean ± SD)	Pre-lecture	15.23 ± 1.92	8.57 ± 2.31	11.77 ± 2.77	35.57 ± 5.22
	Post-lecture	14.53 ± 2.06	8.90 ± 1.97	13.20 ± 2.33^*^	36.63 ± 4.48
Group C (mean ± SD)	Pre-lecture	14.93 ± 2.12	8.33 ± 1.58	11.70 ± 2.82	34.97 ± 4.62
	Post-lecture	15.07 ± 2.23	8.47 ± 1.78	12.27 ± 2.46^*^	35.80 ± 4.78

Compared with pre-lecture scores: ^*^ *p* < 0.05; ^**^ *p* < 0.01. Compared with group B: ^##^ *p* < 0.01. SD, standard deviation.

### Comparison of junior and senior residents’ performance between three groups

One-way ANOVA showed significant difference of post-lecture scoresboth in junior (*p* < 0.0001 for total, *p* = 0.033 for part Ⅰ, *p* < 0.0001 for part Ⅱ, and *p* < 0.0001 for part Ⅲ) and senior residents (*p* < 0.0001for total, *p* < 0.0001forpart Ⅱ, and *p* = 0.017for part Ⅲ) between three groups. Only the part Ⅰ of senior residents showed no significant difference between three groups (*p* = 0.591). Subgroup analysis showed that both junior and senior residents in group A achieved significantly higher post-lecture scores than those in group B (Table 6, *p* < 0.01). Further analysis showed that three parts in junior residents, and part Ⅱ, part Ⅲ in senior residents were significantly improved (Table 6, *p* < 0.01). Besides, the part Ⅰ scores among senior residents were significantly higher in group B, which was consistent with pre-lecture scores (Table 6, *p* < 0.05). There was no significant difference between senior and junior residents in total, part Ⅱ, and part Ⅲ of group B, total and three parts of group C (Table 6).

**TABLE 6:** Comparison of post-lecture scores between junior and senior residents.

	Junior (1st–2nd year, mean ± SD)			Senior (3rd year, mean ± SD)		
	Group A	Group B	Group C	Group A	Group B	Group C
Part Ⅰ	15.89 ± 2.00^**^	14.00 ± 2.25	14.90 ± 2.32	16.18 ± 1.83	15.60 ± 1.07^#^	15.44 ± 2.07
Part Ⅱ	14.00 ± 3.90^**^	9.05 ± 1.96	8.43 ± 1.60	14.73 ± 2.94^**^	8.60 ± 2.07	8.56 ± 2.24
Part Ⅲ	15.58 ± 1.57^**^	13.05 ± 2.52	12.29 ± 2.49	15.27 ± 2.20^**^	13.50 ± 1.96	12.22 ± 2.54
Total	45.47 ± 5.43^**^	36.10 ± 5.00	35.62 ± 4.26	46.18 ± 4.35^**^	37.70 ± 3.16	36.22 ± 6.10

Compared with Group B: ^*^ *p* < 0.05; ^**^ *p* < 0.01. Compared with junior residents: ^#^ *p* < 0.05. SD, standard deviation. SD, standard deviation.

## Residents’ satisfaction

**FIGURE 2:**
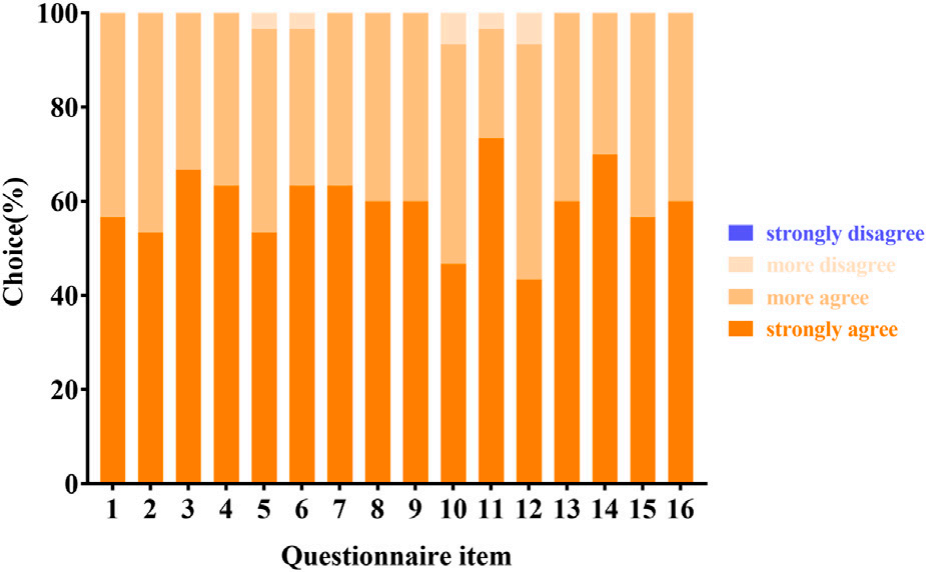
Residents’ feedback to the AI-based PM identification system with a 4-point scale.

All residents in group A responded to the questionnaire. Overall, all respondents were satisfied with the AI-based PM identification system and agreed that the system was helpful, effective, innovative, and beneficial for them to develop the skill of fundus image identification for PM and an extensive understanding of the META-PM classification (Figure 2).

Meanwhile, they also believed that the instructor played an important role in guiding the AI-based PM identification system (Figure 2). We collected residents’ answers to the open-ended question: What are the advantages and disadvantages of the AI-based PM identification system? Many residents confirmed that the AI-based PM identification system benefits them, in terms of efficiency, convenience, innovative design, flexible learning style, self-learning ability, and self-motivation. However, some residents expressed concerns about the hardware requirement, accuracy, and website stability of the AI-based platform.

## Discussion

Standardization is essential for the ophthalmology residency training program. However, significant regional discrepancy among training programs still exist and may result in the variable competency of ophthalmology residents (Wang et al., 2020). AI model showed the potential to provide trainees equal opportunity and minimize the regional difference due to the self-learning model and timely feedback (Fischetti et al., 2022). Particularly, due to the COVID-19 pandemic, routine clinical practice maybe disrupted. Hence, the clinical teaching can also be substantially influenced (Ferrara et al., 2020; Silva et al., 2020). Instead, the AI-assisted education system based on the web platform or mobile devices has broad prospect and has been recommended to make up the theory classes (Pradeep et al., 2021). Moreover, medical students or trainees showed a positive attitude toward AI and high acceptance of the AI-aided medical training (Bisdas et al., 2021). Efforts have been made to develop visual AI courses for further difficult AI courses (Wang et al., 2022) or introductory curriculum into AI in radiology titled AI-RADS for residents education (Lindqwister et al., 2021). This study applied an AI-based PM identification system developed by our team to the residency training program and achieved excellent performance. Our data demonstrated a striking improvement in the residents enrolled in the AI group. This was in consistent with the previous study that AI model could improve the students’ performance in sign and diagnosis part significantly (Wu et al., 2020). Notably, the improvement was significant in total, part Ⅱ, and part Ⅲ while no difference was detected in part Ⅰ. Also, a significant higher scores of part Ⅰ was observed in senior residents compared with junior residents. These results indicated that the PM diagnosis based on typical fundus images can be improved by residents through self-learning or traditional teaching activity. In one of our traditional lecture groups, no difference in total scores was found while part Ⅲ was significantly improved. Part Ⅲ was about the “Plus” lesions detection from fundus images including lacquer cracks, choroidal neovascularization, and Fuchs spot. The improvement in part Ⅲin both traditional and AI training goups indicated that it might be relatively easier for the trainees to identify the “Plus” lesions compared with the mission of categorizing the PM macular lesions according to the META-PM grading system. Overall, the MM classification was the most difficult, followed by the “Plus” lesion detection for residents to fully understand and improve through the didactic lecture solely. In this study, AI model showed better efficiency and great potential help to acquire difficult tasks, shorten the learning curve and training period.

The AI training exhibited better performance as it can provide the huge and multiple fundus images as learning materials in the online database and effectively facilitate the trainees to deepen their understanding of PM signs and morphological features. The database allows the trainees to learn the relevant knowledge efficiently and improve their performance in a short period instead of long-term clinical practice to gain experience. Moreover, our AI system also can archive one-to-many and interactive teaching mode. Due to the merits of efficiency, convenience, flexible learning style, and timely feedback, all residents were satisfied with the system.

Previous study reported the better performance of AI technique in junior residents compared with that of medical students (Han et al., 2022). However, adjunctive tool like ultra-widefield retinal imaging had a better performance in junior residents than senior residents (Lin et al., 2021). Thus, the performance of a new teaching model or adjunct tool may vary during different training stage. Junior and senior residents’ performance even fluctuated during different months of a year (Ali et al., 2022). In our study, both senior and junior residents using AI model achieved significantly higher scores than traditional lecture group, indicating that AI model is worthy for all residents and has great potential for the standardized residency training. However, part Ⅰ in senior residents showed no difference between groups A and B, which may due to the previous clinical training. Given these results, we believe that a well-designed AI-assisted teaching model can provide an effective learning and practice platform to make up the weakness of the traditional teaching mode in ophthalmology residency training program.

The efficiency of traditional lectures by different instructors were also evaluated in this study. The results showed no significant difference in both pre-and post-lecture scores between groups B and C. Subgroup analysis of post-lecture showed significantly higher scores of senior residents compared to junior residents in group B, but not in group C. These results suggested the comparable effect between two experienced instructors only with minor difference and further confirmed the results of the superiority of AI-based teaching mode compared with the traditional lecture.

The free discussion after the lectures was arranged to fully discuss the content of the lecture. However, the discussions between the three groups were highly related to the ir own teaching content of each group with no crosstalk. For example, the traditional lecture group only discussed the relevant content mentioned by the instructor, while the AI-based group only discussed the operating experience of AI platform and the output of the AI platform. Moreover, the results showed no significant improvement in scores of the total, part Ⅰ, and Part Ⅱ after the post-lecture free discussion in traditional lecture group. Given the reasonability and results above, the free discussion after the lectures will not have a significant impact on our results.

Our questionnaire also gave an intact feedback evaluation of our AI-based PM identification system from the residents. All of the participants were satisfied with the AI-based PM identification system and confirmed the positive role of instructor. Comments mainly included efficiency, convenience, innovative design, flexible learning style and higher requirement of hardware.

Compared to previous studies, the present study enrolled more numbers of participants using a new in-house designed AI-based platform. The difference between senior and junior residents was also analyzed. However, the contents regarding the treatment for PM or MM was not included, as the AI model at this stage mainly focuses in the PM identification and MM classification. Further studies are desirable to evaluate the role of AI-based PM identification system on residency training program in more comprehensive respects, including diagnosis, management of disease and even human-machine interaction.

In conclusion, we found the AI-based PM identification system effectively improved the residents’ performance of PM identification, while the group receiving the single traditional lecture showed no significant improvement. Further stratification analysis showed the similar results between the senior and junior residents in both groups, prompting the request for the efficient clinical teaching model to help trainees grasp complex and difficult tasks. Overall, application of the AI-based PM identification system showed advantages in promoting the efficiency of ophthalmology residency training and received positive feedback from residents as well.

## Data Availability

The raw data supporting the conclusions of this article will be made available by the authors, without undue reservation.
